# Maternal lifestyle factors in pregnancy and congenital heart defects in offspring: review of the current evidence

**DOI:** 10.1186/s13052-014-0085-3

**Published:** 2014-11-11

**Authors:** Yu Feng, Di Yu, Lei Yang, Min Da, Zhiqi Wang, Yuan Lin, Bixian Ni, Song Wang, Xuming Mo

**Affiliations:** Department of Cardiothoracic Surgery, The Affiliated Children’s Hospital of Nanjing Medical University, Nanjing, Jiangsu P.R. China; Department of Epidemiology and Biostatistics, Jiangsu Key Lab of Cancer Biomarkers, Prevention and Treatment, Cancer Center, School of Public Health, Nanjing Medical University, Nanjing, Jiangsu P.R. China

**Keywords:** Congenital heart defects, Maternal lifestyle factors, Smoking, BMI

## Abstract

The prognosis of children with congenital heart defects(CHDs) continues to improve with advancing surgical techniques; however, lack of information about modifiable risk factors for malformations in cardiovascular development impeded the prevention of CHDs. We investigated an association between maternal lifestyle factors and the risk of CHDs, because epidemiological studies have reported conflicting results regarding maternal lifestyle factors and the risk of CHDs recently. A review published on 2007 provided a summary of maternal exposures associated with an increased risk of CHDs. As part of noninherited risk factors, we conducted a brief overview of studies on the evidence linking common maternal lifestyle factors, specifically smoking, alcohol, illicit drugs, caffeine, body mass index and psychological factors to the development of CHDs in offspring. Women who smoke and have an excessive body mass index(BMI) during pregnancy are suspected to be associated with CHDs in offspring. Our findings could cause public health policy makers to pay more attention to women at risk and could be used in the development of population-based prevention strategies to reduce the incidence and burden of CHDs. However, more prospective studies are needed to investigate the association between maternal lifestyle factors and CHDs.

## Introduction

Congenital heart defects (CHD) are the most common human birth defects and the leading cause of perinatal mortality, with an incidence of approximately 4 to 50 per 1000 live birth or even higher [1,2]. With the advancing surgical techniques, prognosis of children with complicated and uncomplicated CHDs continues to improve, but the reported incidence remains unchanged [3]. Clinicians and basic scientists have long understood the sources of these cardiovascular developmental errors, and there is wide acceptance of the opinion that that the etiology of CHD is complex and possibly lies within the interaction of environmental exposures and inherited factors [4]. However, lack of enough information about modifiable risk factors for malformations in fetal heart development has impeded the prevention of CHDs.

Although CHDs can occur in the setting of multiple birth defects as part of a syndrome, most are found as isolated defects with no syndromic association. Over the past decade, a multitude of research studies have identified both chromosomal and gene mutations as causation for the syndromic heart malformation [5,6]. While the origin of non-syndromic CHD that accounts for most of all congenital cardiac abnormalities is still under the veil waiting to be further uncovered. Relatively less information has been reported on noninherited factors that may have an adverse effect on the cardiovascular development, which has made it difficult to create population-based strategies to reduce the burden of illness from CHDs and for couples to choose lifestyles to reduce the risk of delivering a child with CHDs.

Recent epidemiologic studies have demonstrated an association between maternal lifestyle factors, specifically smoking, alcohol, illicit drugs use, caffeine use, body mass index and psychological factors and the risk of CHDs in offspring. A review published on 2007 provided a summary of well-known prenatal maternal conditions or exposures associated with an increased risk for cardiac defects [7]. As part of noninherited risk factors, we conducted a brief overview of studies on the evidence linking common maternal lifestyle factors during pregnancy to the development of CHDs in offspring.

### Maternal lifestyle factors and CHD

The studies that reported the association between maternal lifestyle factors during pregnancy and the risk of offspring with CHDs were summarized in Table 1.

**Table 1:** **Maternal lifestyle factors and the risk of offspring with congenital heart defects**

**Maternal lifestyle factors**	**Study design**	**Study period**	**Outcome**	**Exposure**	**OR(95% CI)**	**Reference**
Cigarette smoking	Meta-ananlysis	1971-1999	CHD combined	Smoking	1.07(0.98-1.17)	[8]
	Meta-ananlysis	1959-2010	CHD combined	Smoking	1.09(1.02-1.17)	[9]
	Meta-ananlysis	1971-2011	CHD combined	Smoking	1.11(1.02-1.21)	[10]
			VSD	Smoking	1.44(1.16-1.79)	
	Case–control	1997-2008	CHD combined	Smoking, BMI ≥25 kg/m^2^	2.65(1.20-5.87)	[11]
			Septal defects	Smoking, BMI ≥25 kg/m^2^	2.60(1.05-6.47)	
			Outflow tract anomalies	Smoking, BMI ≥25 kg/m^2^	3.58(1.46-8.79)	
	Case–control	1997-2005	AVSD	Smoking	1.50(1.10- 2.40)	[12]
Alcohol consumption	Case–control	1987-1988	CTD	Drink less than once a week	1.30(1.10-1.90)	[13]
				Drink once a week or more	1.90(1.00-1.34)	
	Case–control	1999-2003	TGA	Drink less than one day per week	2.10(1.10- 3.20)	[14]
	Cohort	1996-2002	VSD	Low-to-moderate levels of alcohol	1.10(0.54- 2.23)	[15]
			ASD	Low-to-moderate levels of alcohol	0.66(0.27- 1.62)	
	Case–control	1996-2005	CHD combined	Binge drinking	2.99(1.19- 7.51)	[16]
	Cohort	1983-2007	ASD	Alcohol Diagnosis	1.36(1.14- 1.63)	[17]
			VSD	Alcohol Diagnosis	1.77(1.27 2.46)	
	Cohort	2002-2010	CHD combined	Alcohol use	1.90(1.70- 2.00)	[18]
	Case–control	1999-2005	CHD combined	Drink several times a week	1.73(0.52- 5.70)	[19]
				Drink several times a month	1.34(0.79-2.24)	
Illicit drug use	Case–control	1968-1980	VSD	Cannabis use	2.35(1.43- 3.86)	[20]
				Cannabis use for three or more days per week	3.73(1.56- 8.96)	
	Case–control	1981-1989	VSD	Cocaine	2.99(1.69-5.30)	[21]
	Case–control	1981-1989	Single ventricle	Marijuana	0.90(0.10- 6.90)	[22]
Caffeine Use	Systematic review	1966-2004	CHD combined	Coffee	1.10(0.90- 1.50)	[23]
				Tea	1.00(0.90-1.20)	
	Case–control	1996-1998	CHD in Down syndrome	Any caffeine	0.89(0.42-1.88)	[24]
				Coffee	0.75(0.39-1.44)	
				Tea	1.11(0.60-2.03)	
				Cola	0.69(0.38-1.27)	
				Cocoa	3.95(0.81-19.2)	
	Case–control	1991-1993	CHD in Down syndrome	≥4 cups of coffee vs. <4 cups	1.50(0.70-3.30)	[25]
BMI	Meta-ananlysis	1996-2008	CHD combined	Obesity	1.30(1.12-1.51)	[26]
			Septal anomalies	Obesity	1.20(1.09-1.31)	
			TOF	Obesity	1.10(0.76-1.61)	
			TGA	Obesity	1.41(0.97-2.06)	
	Meta-ananlysis	1969-2012	CHD combined	Overweight	1.08(1.02-1.15)	[27]
				Moderate obesity	1.15(1.11-1.20)	
				Severe obesity	1.39(1.31-1.47)	
			TOF	Severe obesity	1.94(1.49-2.51)	
			AVS	Underweight	1.47(1.01-2.15)	
	Case–control	2004-2009	PAS	Obesity	1.36(1.17-1.58)	[28]
			TOF	Obesity	1.62(1.30-2.02)	
			TGA	Obesity	1.45(1.16-1.80)	
			VSD	Obesity	1.15(1.07-1.23)	
Psychological factors	Case–control	1978-2008	CHD combined	Bereavement	1.11(1.00-1.22)	[29]
	Case–control	2004-2005	CHD combined	Mental stress	3.93(1.94-7.94)	[30]
	Case–control	1996-2005	CHD combined	High stress level vs. low stress level	0.74(0.45-1.22)	[25]

Abbreviation: CHD, congenital heart defect; VSD, Ventricular Septal Defect; ASD, Atrial Septal Defect; TOF, Tetralogy of Fallot; TGA, Transposition of the Great Arteries; AVSD, Atrioventricular Septal Defect; CTD,Conotruncal defects; PVS, Pulmonary valve stenosis; COA, Coarctation of the aorta; AVS, Aortic valve stenosis; PAS, pulmonary artery stenosis.

### Evidence of maternal smoking during pregnancy and CHD

A study by Alberman et al. [31] was one of the first to report the association between maternal cigarette smoking and CHDs. However, the evidence since then has been mixed, with some studies showing positive associations and others providing null results. To date, a total of three meta-analyses have investigated the association between maternal smoking during pregnancy and CHDs in offspring. The first meta-analysis published on1999 found no association for all CHDs combined (OR, 1.07; 95% CI, 0.98-1.17) and mixed results for analyses of specific groups or phenotypes [8]. The second meta-analysis by Hackshaw et al. [9] estimated the effects of maternal smoking across a spectrum of birth defects including heart defects (OR, 1.09; 95% CI, 1.02-1.17). However, the study did not evaluate the effects of maternal smoking on CHD subtypes, and dose–response relationships (i.e., increasing levels of smoking) were not assessed. A latest meta-analysis [10] of studies published between 1971 and 2011 (23 case–control studies, 5 cohort studies, and 5 cross-sectional studies) found positive association for all CHDs combined (RR, 1.11; 95% CI, 1.02-1.21). According to analysis, as for specific groups, women who smoked during pregnancy were more likely to give birth to a child with 12 of 17 CHD subtypes analyzed compared with nonsmoker. The highest risk was for septal defects as a group (RR, 1.44; 95% CI, 1.16-1.79). After that, a recent study reported that only valvar pulmonary stenosis(VPS) was highly associated with mothers who smoked 20 cigarettes or more per day (*P* = 0.03). When both mother and father consumed at least 20 cigarettes per day, VPS and coarctation of the aorta would have a significant p-value (0.03 and 0.02 respectively) [32]. Another study reported as opposed to smoking or high BMI alone, the risk for CHD in the offspring of women with high BMI (≥25 kg/m^2^) who also smoked was significantly increased. The adjusted OR was 2.65 (95% CI, 1.20-5.87) for all CHD, 2.60 (95% CI, 1.05- 6.47) for septal defects and 3.58 (95% CI, 1.46- 8.79) for outflow tract anomalies [11]. Recent analyses of small case groups based on the National Birth Defects Prevention Study(NBDPS) data have identified associations between maternal smoking and atrioventricular septal defects (AVSDs) [12].

The different results for analyses of specific groups of CHDs probably reflect methodological limitations, and other environmental factors. Further research based on large population using standardized ascertainment of case and classification methods is needed to determine whether there is an association between maternal smoking and the risk of CHDs in offspring.

### Evidence of maternal alcohol consumption during pregnancy and CHD

Ever since the first description of the fetal alcohol syndrome by Jones and Smith in 1973 [33], many observational studies have been published on the topic of alcohol consumption in pregnant women and the effects on the development of their fetus and child, including cardiac defects. Recently, a total of seven studies investigate the association between maternal alcohol consumption during the pregnancy and CHDs. A population-based case–control study of California births indicated that compared with nonconsumers, women who consumed alcohol less than once a week had a 1.3-fold higher risk of having infants with a conotruncal heart defect (95% CI, 1.00- 1.90), and women who consumed alcohol once a week or more had a 1.9-fold increase in risk (95% CI, 1.00- 3.40) [13]. In another population-based case–control study among a cohort of California births between July 1999 and June 2003, maternal consumption of alcohol less than one day per week was associated with a 2.1-fold increased risk of d-transposition of the great arteries (95% CI, 1.10- 3.20) [14]. According to a cohort study including 80,346 pregnant women enrolled into the Danish National Birth Cohort, exposure to low-to-moderate levels of alcohol on a weekly basis or occasional binge drinking during pregnancy was not statistical significantly increased risk of isolated ventricular septal defect(VSD) and atrial septal defect(ASD) in offspring [15]. For binge drinking, a case–control study in the Pregnancy Risk Assessment Monitoring System (PRAMS) showed significantly increased risk of CHDs among mothers who reported binge drinking more than once compared to mothers who did not report binge drinking in the 3 months prior to pregnancy (OR, 2.99; 95% CI, 1.19-7.51) [16]. Two more recent cohort studies in Canada and Australia reported a significant association between maternal alcohol consumption during pregnancy and overall CHDs (OR, 1.90; 95% CI, 1.70- 2.00) or ASD (OR, 1.36; 95% CI, 1.14- 1.63) and VSD (OR, 1.77; 95% CI, 1.27- 2.46), respectively [17,18]. However, other studies have failed to identify an association [19].

### Evidence of maternal illicit drug use during pregnancy and CHD

Illicit drug use was recorded for marijuana, hashish, cocaine, hallucinogens, heroin, and methadone. Marijuana and hashish use were combined to reflect cannabis exposure. A case–control study in Atlanta reported maternal cannabis use, according to self- and proxy-report, was associated with a two-fold increased risk of VSD. Self-reported cannabis use for fewer than three days per week resulted in a similar OR, while use three or more days per week resulted in an almost four-fold increased risk (OR, 3.73; 95% CI, 1.56- 8.96) [20]. A case–control study using data from the Baltimore-Washington Infant Study (BWIS) examined the association between maternal cocaine use and isolated membranous ventricular septal defects (VSD). An OR of 2.99 for maternal cocaine use dropped to 2.15 when taking into account paternal cocaine use (a change of 28%) and to 2.52 when paternal marijuana use was considered (a change of 16%). Although maternal cocaine use was an effect modifier for paternal cocaine use, it was not statistically significant as an independent risk factor [21]. Another case–control study of single ventricle using data from the BWIS reported no association between maternal marijuana and single ventricle in infants (OR, 0.90; 95% CI, 0.10-6.90) [22]. Further studies are warranted to elucidate the possible association of illicit drug use with CHDs in offspring.

### Evidence of maternal caffeine use during pregnancy and CHD

Caffeine is a natural component of coffee, tea, cocoa, and cola products. It is teratogenic in animal studies when administered at high concentrations [34]. In humans, caffeine and its metabolites easily cross the placenta and reach the fetus [24]. Most studies on the human teratogenicity of caffeine have not shown an important effect. In a systematic review published on 2006 [23] investigating the association between maternal exposure to caffeine and risk of congenital anomalies, slight elevations were observed for associations between coffee intake and cardiovascular malformations (OR, 1.10; 95%CI, 0.90-1.50), but not for the association between tea and cardiovascular malformations (OR, 1.00; 95%CI, 0.90-1.20). Fixler and colleagues observed an OR of 0.75 (95% CI, 0.39-1.44) for the occurrence of cardiac defects with coffee consumed daily and an OR of 0.89 (95% CI, 0 .42-1.88) for consumption of caffeine in general [24]. In a case–control study which evaluated risk factors present during early pregnancy in a multiracial population of 687 infants with Down syndrome, the proportion of mothers who drank four or more cups of coffee daily (4.2%) was too small to analyze high caffeine intake from either tea or soft drinks [25].

### Evidence of maternal body mass index during pregnancy and CHD

Nowadays, obesity rates have an increasing tendency, since the incidence of obesity in both developed and developing countries is still rising over the years. Epidemiologic data from the National Health and Nutrition Examination Survey described that, from 2007–2008, 28-32% of childbearing-aged women were obese and that 7.2-8.4% of them were morbidly obese (BMI ≥ 40 kg/m^2^) [35]. From a public health perspective, recent studies have highlighted the increased risks that are associated with obesity in pregnancy and have appealed for optimal treatment of the pre gravid obese women. Numerous studies have shown that obese women appear to be at a higher risk of pregnancy complications, such as preeclampsia, gestational diabetes mellitus, preterm delivery, and cesarean delivery [36-39], as well as adverse fetal and neonatal outcomes, such as congenital heart defect. A meta-analysis of 18 studies published between 1966 and 2008 found an association of maternal obesity with an increased risk of cardiovascular anomalies (OR, 1.30; 95% CI, 1.12-1.51) and septal anomalies (OR, 1.20; 95% CI, 1.09-1.31) [26]. A recent meta-analysis of 14 epidemiological studies [27] demonstrated an association between overweight, moderate obesity, and severe obesity and all CHD combined (OR, 1.08, 95% CI, 1.02-1.15; OR, 1.15, 95% CI, 1.11-1.20; and OR, 1.39, 95% CI, 1.31-1.47, respectively) as well as some individual defects such as pulmonary valve stenosis, hypoplastic left heart syndrome, and outflow tract defects, with the highest risk of tetralogy of Fallot for obese mothers for (OR, 1.94; 95% CI, 1.49-2.51). Being underweight did not increase the risk of any of the aforementioned CHDs but did increase the risk of aortic valve stenosis (OR, 1.47; 95% CI, 1.01-2.15). In a more recent case–control study that evaluated the risk of congenital anomalies with different doses of maternal pre-pregnancy obesity in Florida, five CHD phenotypes showed evidence of a dose–response pattern with increasing severity of maternal obesity corresponding to increased odds of each defect [28]. In case–control studies, obesity is a complex condition that should to be studied carefully to minimize underreporting of body weight. We also should take into account the possibility of confounding by other factors associated with nutrition, such as the use of multivitamin supplements or intake of micronutrients.

### Evidence of maternal psychological factors and CHD

Maternal stress is measured by maternal reports of divorce, separation, job loss, or death of a close relative or friend. A large registry-based study reported maternal emotional stress may be a risk factor for CHDs in infants, using bereavement around conception as an indicator of maternal exposure to stress (OR =1.11, 95% CI 1.00–1.22). The association was most for infants of mothers who had lost a partner or child (OR =1.32, 1.04–1.67) [29]. Maternal mental stress during early pregnancy was significant associated with aggregate cardiac defects in a hospital-based case–control study of 164 patients with CHDs (OR, 3.93; 95% CI, 1.94–7.94) [30]. No increased risk of major cardiac malformations among offspring was seen of women who had high stress level compared with low stress level in a case–control study of 237 cases [16].

## Discussion and conclusion

The reviewed studies indicate that maternal lifestyle factors during pregnancy may contribute to congenital heart defects in the offspring. However, because of differences in methods, these studies are only suggestive. The majority of studies found an association between exposure to tobacco smoking during pregnancy and infant’s risk of CHDs, but not all subtypes of CHDs. The results for alcohol exposure were also inconsistent, and some papers included in this review only showed a statistically significant association with specific groups or phenotypes. Only half of the papers showed a statistically significant association between maternal illicit drug use during pregnancy and CHD. Most studies showed that increasing maternal body mass index was associated with an increased risk of CHDs; severe obesity was an even greater risk factor for the development of CHDs. However, the results for studies about maternal caffeine use and psychological factors were mixed.

In interpreting findings on possible associations between maternal lifestyle factors and CHDs, we must remember that such associations from observational studies may be due to the exposures or factors of interest, but they may also be a result of chance, bias, or confounding. An observational study can yield an association as a result of sampling variation of the controls or multiple comparisons in an exploratory study. Since the assessment of exposure to many factors is often based on parental recall after the birth of the child, recall bias should be concerned [7].

The mechanisms by which maternal lifestyle factors may result in CHDs still remain unknown. Findings have shown that maternal smoking has adverse effects on the developing fetus, including hypoxia caused by carbon monoxide, nicotine, and reduction in the supply of essential nutrients to the embryonic tissues [40,41]. Additionally, polycyclic aromatic hydrocarbons, common components of cigarette smoke, are suspected teratogens in laboratory animals and humans [42,43]. The mechanisms by which alcohol consumption may result in CHDs remain to get confirmed, even though its etiology has been the focus of much study, especially the cellular and molecular mechanisms [44-46]. Additionally, findings have shown that alcohol consumption during pregnancy may affect the Wnt/β-catenin signaling which allows normal gene activation and cardiogenesis [47]. Cell death is a hypothesized mechanism for muscular VSD formation and alcohol exposure has been shown to result in abnormal cell development and cell death [48]. Cocaine or marijuana, a vasoconstrictor, has been investigated as a potential teratogen because exposure may result in vascular disruptions and hypoperfusion. Some studies among humans have reported intake of caffeine or coffee could induce increased levels of homocysteine and decreases in insulin sensitivity [49]. Vascular disruption, increased homocysteine levels, and oxidative stress associated with hyperglycemia are potential mechanisms for various congenital malformations [50-52]. For the same reason, women who are obese might have diabetes mellitus, which appears to be an important pathogenetic factor that is associated with a wide spectrum of CHDs [53]. Maternal stress could affect fetal development by several plausible mechanisms, such as catecholamine production and corticosteroid production, which may be associated with the development of birth defects. Another potential mechanism is that stress may lead to harmful coping behaviors (e.g., increased substance use or cigarette smoking or decreased dietary quality) and therefore affect development indirectly.

In conclusion, this review summarizes the current state of knowledge of maternal lifestyle factors that may increase the likelihood of congenital heart defects in offspring. To date, no public policies or interventions are specifically directed at reducing the public health impact of congenital heart defects [7]. Our findings could cause public health policy makers to pay more attention to at-risk populations and could be used in the development of population-based prevention strategies to reduce the incidence and burden of CHDs, especially women who smoke during pregnancy and have an excessive BMI. However, more prospective studies are needed to further investigate the association between maternal lifestyle factors and CHDs, especially with regard to the different subtypes of CHDs.

## Abbreviations

CHD: Congenital heart defect
BMI: Body mass index
OR: Odds ratio
CI: Confidence interval
VSD: Ventricular septal defect
ASD: Atrial septal defect
TOF: Tetralogy of fallot
TGA: Transposition of the great arteries
AVSD: Atrioventricular septal defect
CTD: Conotruncal defects
VPS: Valvar pulmonary stenosis
COA: Coarctation of the aorta
AVS: Aortic valve stenosis
PAS: Pulmonary artery stenosis
